# Complete Genome Sequencing of the Divergent Guiana Dolphin Morbillivirus (GDMV), Brazil

**DOI:** 10.3390/v17040582

**Published:** 2025-04-18

**Authors:** Kátia Regina Groch, Sueli Akemi Taniwaki Miyagi, Josué Díaz-Delgado, Elitieri B. Santos-Neto, José Lailson-Brito, Paulo Eduardo Brandão, José Luiz Catão-Dias

**Affiliations:** 1 Department of Pathology, School of Veterinary Medicine and Animal Sciences, University of São Paulo, São Paulo 05508-207, Brazilzecatao@usp.br (J.L.C.-D.); 2 Department of Preventive Veterinary Medicine and Animal Health, School of Veterinary Medicine and Animal Sciences, University of São Paulo, São Paulo 05508-207, Brazil; 3 Laboratório de Mamíferos Aquáticos e Bioindicadores (MAQUA), Faculdade de Oceanografia, Rio de Janeiro State University (UERJ), Rio de Janeiro 20550-013, Brazil; elitieri.neto@gmail.com (E.B.S.-N.);

**Keywords:** cetacean morbillivirus, cell culture, virus isolation, marine mammal stranding, mortality outbreak, epizootics, *Sotalia guianensis*

## Abstract

Cetacean morbillivirus (CeMV) is a major threat to cetaceans worldwide, causing individual deaths and outbreaks of mass mortality. Based on partial sequences of the viral phosphoprotein, CeMV is subclassified into seven strains and two distinct lineages. To date, only CeMV-1 strains, including the dolphin morbillivirus (DMV), have been completely sequenced. The CeMV-2 lineage was first reported in Guiana dolphins (*Sotalia guianensis*) in Brazil and was associated with an unusual mortality event in 2017–2018. Here we provide the nearly complete Guiana dolphin morbillivirus (GDMV) genome sequence, representing the first within the CeMV-2 lineage. GDMV was isolated using Vero.DogSLAMtag cells, the viral RNA was extracted, and deep sequencing analysis was performed. Gaps in the viral genome were completed by Sanger sequencing. The final genome length was 15,607 nucleotides covering 99.3% of the DMV reference genome, including full sequences of the six structural proteins encoded by morbillivirus. The sequence similarity was 74–77.9% to other CeMV strains, with highest identity to the DMV. The complete L protein amino acid sequence comparison-based taxonomy indicates that GDMV is a distinct morbillivirus species; however, as GDMV and CeMV-1 strains infect a similar host spectrum, our findings support that GDMV represents a new CeMV-2 lineage.

## 1. Introduction

Cetacean morbillivirus (CeMV; *Morbillivirus ceti*) is a member of the genus *Morbillivirus*, family *Paramyxoviridae*, which includes important animal and human pathogens that result in severe and often fatal diseases. Morbilliviruses are enveloped viruses characterized by the presence of non-segmented, linear, single-stranded negative-sense RNA genomes, with circa 15,040 (representative rodent morbillivirus: longquan berylmys bowersi morbillivirus 1, LBbMV) to 16,050 (feline morbillivirus, FeMV) nucleotides in size, which encode eight proteins: N (nucleocapsid), P (phosphoprotein), V, C, M (matrix), F (fusion), H (hemagglutinin), and L (large polymerase), six of which are structural and present in the virion [1]. Morbilliviruses affect mammals, causing measles in humans, peste des-petits-ruminants in small ruminants, rinderpest in cattle, phocine distemper in seals, and canine distemper in carnivores [2].

Cetacean morbillivirus is one of the major threats to cetaceans. Several strains of CeMV have been detected in an increasing number of species in different geographic locations worldwide, causing individual deaths and outbreaks of mass mortality. Based on partial sequences of the viral P gene, CeMV has been subclassified into seven strains and two distinct CeMV lineages: CeMV-1, which includes dolphin morbillivirus (DMV), first detected in striped dolphins (*Stenella coeruleoalba*) in the Mediterranean Sea [3]; porpoise morbillivirus (PMV) in Harbor porpoises (*Phocoena phocoena*) in Northern Ireland [4]; pilot whale morbillivirus (PWMV) in a long-finned pilot whale (*Globicephala melas*) in New Jersey, USA [5]; and beaked whale morbillivirus (BWMV) in a Longman’s beaked whale (*Indopacetus pacificus*). The CeMV-2 lineage comprises the Guiana dolphin morbillivirus (GDMV), first detected in a Guiana dolphin (*Sotalia guianensis*) in Brazil [6], and the strain found in an Indo-Pacific bottlenose dolphin (*Tursiops aduncus*), detected in western Australia (IPDMV) [7]. Recently, another divergent strain, the Fraser’s dolphin morbilliviurs (FDMV), has been detected in a Fraser’s dolphin (*Lagenodelphis hosei*) in Hawaii [8].

To date, DMV, PMV, and PWMV, all from the CeMV-1 lineage, are the only strains with their genomes completely sequenced. Similar to other Morbillivirus members, the DMV genome was determined to be about 15.7 kb long, with six genes encoding eight proteins, six of which are structural, and they are organized in the following order: 3′ nucleoprotein (N)—phosphoprotein (P)—matrix protein (M)—fusion protein (F)—hemagglutinin protein (H)—large protein (L) 5′ [9].

The diagnosis of CeMV in stranded cetaceans is challenging and often relies on viral detection by reverse transcription, followed by polymerase chain reaction (RT-PCR) and/or immunohistochemistry (IHC) coupled with histopathologic changes. PCR targeting small fragments of the conserved gene regions of N, P, F, and L genes is the most commonly applied method for CeMV detection [10,11,12,13,14]. Viral isolation in cell cultures is regarded as the gold-standard method for definitive diagnosis of viral infection; however, the propagation and isolation of wild strains are difficult, and the sensitivity is low. Previous studies indicated that African Green Monkey Kidney (Vero) cell line may be suitable for CeMV isolation [4,15], and the Vero cell line expressing the Canine Signaling Lymphocyte Activation Molecule (SLAM/CD150) is more permissive for DMV replication [16]. More recently, DMV and PMV have been successfully isolated from archival tissue samples using Vero.DogSLAMtag cell cultures, obtaining complete viral genomes utilizing next generation sequencing (NGS), completing gaps by Sanger sequencing, and obtaining genome termini by RACE (Rapid Amplification of cDNA Ends) protocol [17,18].

The GDMV strain was first discovered in a stranded Guiana dolphin in 2010 [6]. In late 2017 and early 2018, an unusual mortality event of Guiana dolphins due to GDMV claimed at least 277 dolphins in Rio de Janeiro, Brazil [19,20,21]. We have compelling evidence that GDMV is endemic and widespread along the Brazilian coast and affects multiple cetacean species, including Southern right whales (*Eubalaena australis*) [22], humpback whales (*Megaptera novaeangliae*) [23,24,25], killer whales (*Orcinus orca*) [26], and Atlantic spotted dolphins (*Stenella frontalis*) [25]. Partial sequences of the N gene, P gene, and L gene indicate that GDMV is one of the most divergent CeMV strains [6,17,19,20]. In this study, we report the successful isolation of GDMV from a Guiana dolphin stranded in Sepetiba bay, Rio de Janeiro, Brazil in 2017, representing the 2017–2018 mortality outbreak. This virus was isolated in Vero.DogSLAMtag cell culture, and GDMV’s genome sequence was almost fully characterized, representing the first of the CeMV-2 lineage, and reported in this paper.

## 2. Materials and Methods

### 2.1. Material

The Guiana dolphin was a juvenile male found dead in Sepetiba Bay along the Rio de Janeiro coast, Brazil, on 23 December 2017. Necropsy was performed, and the results have been published as case no. 15 in Groch et al. [20] and case no. 5 in Díaz-Delgado et al. [27,28]. In brief, the dolphin’s nutritional condition was moderate, and the main pathologic findings included diffuse lymphoid depletion, bronchointerstitial pneumonia, and urothelial intracytoplasmic viral inclusions in the urinary bladder, suggestive of CeMV infection. Additionally, there was verminous pleuropneumonia by *Halocercus brasiliensis*, necrotizing hepatitis with intralesional *Toxoplasma gondii*, acute cerebral hemorrhage and perivascular edema, ulcerative gastritis, acute intestinal hemorrhage, and multifocal renal tubular degeneration and necrosis. GDMV infection was detected using real-time RT-PCR in the lungs, pulmonary lymph nodes, and brain, and through IHC in the lungs, trachea, tongue, intestine, liver, lymph nodes, spleen, kidney, urinary bladder, and epidermis. *Toxoplasma gondii* was detected by PCR in the brain [20].

### 2.2. Viral Isolation

A frozen pulmonary lymph node sample of the Guiana dolphin was processed for virus isolation using Vero.DogSLAMtag’s cell line—kindly provided by Dr. João Pessoa Araújo (UNESP—São Paulo State University, Botucatu, São Paulo, Brazil)—utilizing standard virological procedures. In short, approximately 300 mg of the tissue sample was cut with a scalpel and disrupted with a syringe needle in 3 mL of Minimum Essential Medium (MEM, Thermo Fisher Scientific, Waltham, MA, USA) in a 15 mL tube, submitted to thermal shock twice (frozen in liquid nitrogen for 15 s and incubated at 56 °C for 10 min), and clarified by centrifugation (12,000× *g* for 5 min at 4 °C). The supernatant was filtered through disposable filters with a 0.45 µm pore diameter, and 1 mL was inoculated onto subconfluent monolayer Vero.DogSLAMtag cell cultures. To allow viral adsorption, the flask was incubated for 60 min at 37 ± 1 °C with 5% CO_2_ and gentle rocking every 15 min. Mock-infected cell cultures were used as negative controls. One mL of MEM with 2% fetal bovine serum (FBS) was dispensed in each flask, and the cells were incubated at 37 ± 1 °C with 5% CO_2_ for a maximum of 4 days. Cultures were inspected daily under a microscope for the occurrence of cytopathic effect (CPE). Re-inoculation of the flasks showing at least 80% of CPE or blind passage from flasks not exhibiting CPE were carried out until an overall amount of three serial passages was achieved. For re-inoculation, samples either showing or not showing CPE were harvested by freezing-thawing and sub-cultured onto fresh cells and incubated for a further 4 days in the same conditions as described above.

### 2.3. Immunohistochemical Analysis

CPE-positive and mock-infected cell subcultures were performed on chamber slides for IHC analysis. A monoclonal IgG2B (kappa light chain) antibody against the nucleoprotein antigen of canine morbillivirus (1:200 dilution; CDV-NP MAb, VMRD Inc., Pullman, WA, USA) was used as the primary antibody. Sections of lymph nodes from a GDMV-positive Guiana dolphin were used as positive control. For negative controls, sequential sections of the positive control tissues were incubated with nonimmune homologous serum instead of the primary antibody. Slides with and without CPE were rinsed in phosphate-buffered saline (PBS, pH 7.4) and fixed in 10% neutral buffered formalin for 10 min, washed in PBS, and incubated in C_3_H_6_O for 20 min at 4 °C. The IHC reagents and procedures were performed as described [20].

### 2.4. RNA Extraction and Deep Sequencing Analysis

Cells were harvested by freezing-thawing, and 233 μL of the supernatant was treated with Turbo™ DNase (Ambion Inc., Austin, TX, USA) and RNase™ Cocktail Enzyme mix (Ambion) according to the manufacturer’s instructions. RNA isolation was carried out using a QIAamp^®^ cador^®^ Pathogen Mini Kit (QIAGEN Sciences LLC, Germantown, MD, USA) without carrier RNA according to manufacturer instructions. A total of 10 µL of total RNA was reverse-transcribed to ss-cDNA employing the SuperScript III Reverse Transcriptase (Invitrogen, Thermo Fisher Scientific, Waltham, MA, USA) with 50 ng/μL of random primers. The ss-cDNA was tested by real-time PCR to semi-quantitatively assess the viral concentration, employing primers Forward GD-F: 5′-GCAGTTATGATCCCGGAAGA-’3 and Reverse GD-R: 5′-TTGAGATTGGGATCCAGAGG-’3 (GD qPCR assay) and a PowerUp^TM^ SYBR^®^ Green RT-PCR kit (Thermo Fisher Scientific, Waltham, MA, USA) as previously described [14]. Ds-cDNA was synthesized using 3′-5′ exo—Klenow DNA Polymerase™ (Ambion), purified with magnetic beads (AMPure XP, Ambion), quantified using a Qubit dsDNA BR Assay kit on the Qubit 3.0 Fluorometer (Thermo Fisher Scientific), and diluted to 0.2 ng/µL for downstream application of the Nextera XT DNA Library Prep Kit protocol (Illumina, San Diego, CA, USA). Libraries were submitted to massive parallel sequencing on a NextSeq500 platform (Illumina) in paired-end mode (2 × 300 bp) loading a MiSeq Reagent Kit v2 Nano 300 cartridge (Illumina). Reads were assembled de novo using CLC Genomics Workbench 10.1.1 (QIAGEN).

### 2.5. Primer Design for Genome Gaps Closure

To close genome gaps, nine new primers based on the partial GDMV sequence from this study were designed using Primer3Plus version 3.3.0 to perform conventional RT-PCR. One single primer (GDMV-1R) was designed based on a previously published GDMV sequence [20]. The primers were designed to have a melting temperature (Tm) between 43 and 60 °C and to not form hairpin loops or primer dimers. The NCBI Primer-BLAST tool (Primer3 version 2.5.0) was used to confirm the specificity of the primers for CeMV. In addition, a single primer (DMV-1F) [29] and a primer pair (CDV-3F, CDV-4R) [30], previously described, were used. In total, 13 primers were used (Table 1).

### 2.6. Conventional RT-PCR

Total RNA was extracted from 250 μL of cell culture supernatant by using TRIzol Reagent (Invitrogen) according to the manufacturer’s instructions. The synthesis of cDNA was carried out in a 20 μL reaction using random primers and an iScript™ cDNA Synthesis Kit (Bio-Rad Laboratories, Inc., Hercules, CA, USA). The RT-PCR was performed in a C1000 Touch thermal cycler (Bio-Rad Laboratories) with a 25 μL reaction mixture containing 250 nM of forward and reverse primers, 1× OneTaq^®^ Quick-Load^®^ Master Mix with Standard Buffer (New England Biolabs, Evry, France), and 2 μL of cDNA. The primer combinations and annealing temperatures are specified in Table 2. The RT-PCR conditions were as follows: initial denaturation for 30 s at 94 °C, 40 cycles of amplification (30 s at 94 °C, 60 s at annealing temperature, and 60 s at 68 °C), and a final extension step at 68 °C for 5 min. The resulting PCR products were analyzed by electrophoresis on 1.5% agarose gel containing SYBR Safe DNA Gel Stain (Invitrogen). The PCR products of positive samples were purified by using a QIAquick PCR Purification Kit (QIAGEN Sciences LLC) and directly sequenced from both ends by an ABI PRISM 3100 Genetic Analyzer using a BigDye Terminator v.3.1 cycle sequencing kit (Applied Biosystems, Thermo Fisher Scientific, Waltham, MA, USA). Frozen lung samples from a GDMV-positive Guiana dolphin were used as positive control. Nuclease-free water instead of sample was included as negative control. The newly generated GDMV genome was deposited in GenBank under Acc. no. PV298625.

### 2.7. Phylogenetic Analysis

Multiple sequence alignment was performed with MEGA7 [31] using Clustal W. Sequence similarities were calculated using p-distance. The estimates of evolutionary divergence between sequences were conducted including 28 complete genome nucleotide sequences and a total of 16,618 positions. The best nucleotide substitution model was evaluated using MEGA7 and was used for phylogeny inference according to the maximum likelihood (ML) criterion [31], which was General Time Reversible with Gamma distribution and Invariant sites (GTR + G + I). The robustness of the hypothesis was tested in 1000 non-parametric bootstrap interactions.

The phylogenetic analysis of complete L protein amino acid sequences was aligned by Clustal W with gap generation penalties of 5 and extension penalties of 1 in both multiple and pairwise alignments. The evolutionary history was inferred by using the Maximum Likelihood method and JTT matrix-based model. The tree was drawn to scale, with branch lengths measured in the number of substitutions per site. The neighbor-joining method based on p-distance was used for the phylogenetic analysis of complete N, P, M, F, H, and L protein amino acid sequences.

## 3. Results

**Figure 1:**
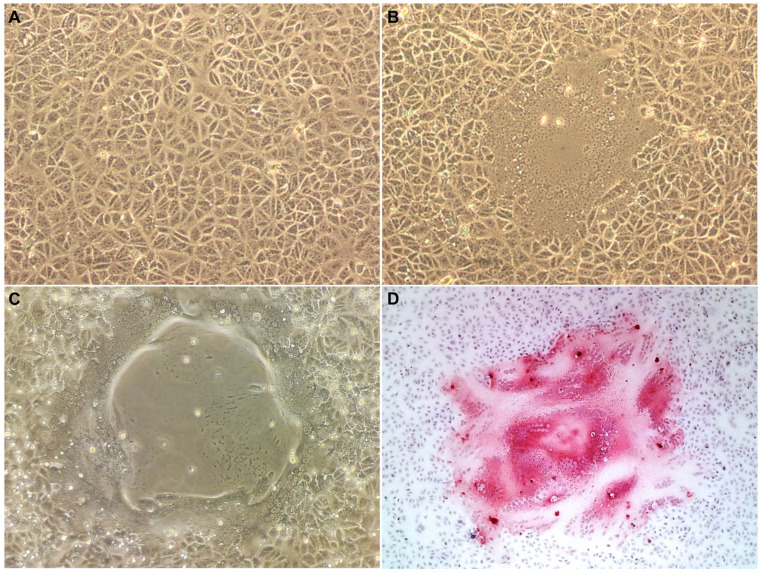
(**A**) Vero/dog SLAM, mock infected cells, 10×, 40 h; (**B**) Vero/dog SLAM, syncytium. 10×, 40 h post infection (p.i.); (**C**) Vero/dog SLAM, syncytium and central area of cell loss surrounded by syncytia; 4×, 96 h p.i.; (**D**) Immunohistochemistry for morbillivirus using an antibody from canine distemper virus nucleoprotein. There is strong positive nuclear and cytoplasmic immunolabeling of Vero/dog SLAM cells syncytia. 4×; 96 h p.i.

Successful viral isolation on the Vero.DogSLAMtag cell line was demonstrated by syncytia formation 24 h after inoculation (first and second cell passages). Up to 93 foci of CPE, consisting of multifocal syncytia formation and coalescing central areas of cell loss, were observed at 72 h after inoculation on the second cell passage. Strong, diffuse, positive cytoplasmic and nuclear immunolabeling was observed in the syncytia (multinucleated cells) in areas of CPE (Figure 1). GDMV propagation was then confirmed by real time RT-PCR positivity in the supernatant.

De novo assembly using CLC Genomics Workbench v. 10.1.1 resulted in five GDMV contigs, with a contig length ranging from 1274 to 5453 nucleotides (nts) and coverages ranging from 27 to 43. Based on the DMV reference genome, the gaps ranged from 52 to 985 nts and were filled by consensus sequences obtained with 6 conventional RT-PCRs followed by 3 nested PCRs. All of the RT-PCR reactions gave the expected amplicons and were successfully directly sequenced. The final consensus length obtained was 15,607 nts, covering 99.3% of the DMV reference genome and extending from nucleotide 108 to 15,708. The alignment between the sequence generated for the GDMV isolate (hereafter named GDMV_SG_2017) and the reference sequence showed that the genomic regions not covered by the sequence corresponded to 107 putative nts of the viral RNA 3′ leader region.

**Figure 2:**
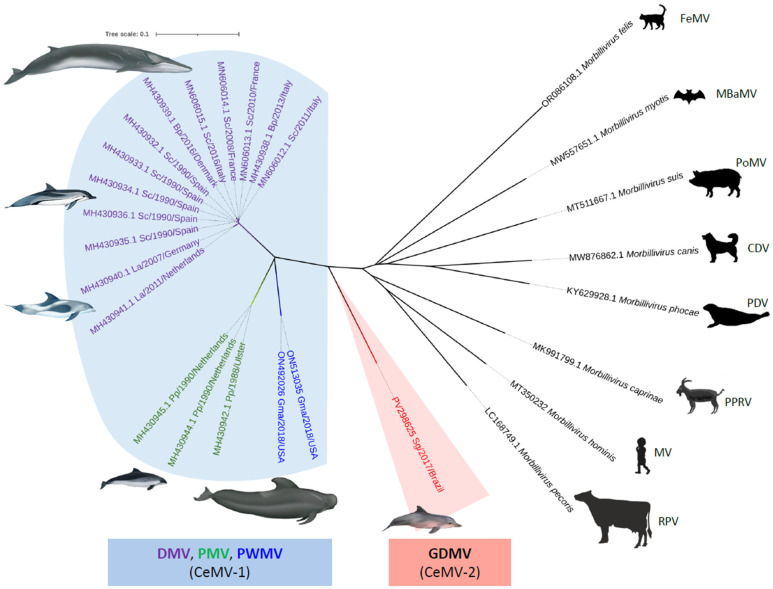
Unrooted phylogenetic tree showing complete genome nucleotide sequences of the cetacean morbillivirus (CeMV) isolate found in a stranded Guiana dolphin (*Sotalia guianensis*) from Rio de Janeiro, Brazil, 2017, and those of other previously described morbilliviruses. The phylogram was generated by the neighbor-joining method, bootstrap of 1000 replicates, with a total of 15,020 bp. The tree is drawn to scale, with branch lengths and the evolutionary distances computed using the p-distance method. For comparison, recognized CeMV strains were included when available. CeMV-1 and CeMV-2 represent the two distinct CeMV lineages. The sequence names include GenBank accession number, species of cetacean, year of stranding, and location. The scale bar indicates nucleotide substitutions per site. Abbreviations for each part of the sequence name are as follows: Bp, *Balaenoptera physalus*; Gma, *Globicephala macrorhynchus*; La, *Lagenorhynchus albirostris*; Pp, *Phocoena phocoena*; Sc, *Stenella coeruleoalba*; Sg, *Sotalia guianensis*. Abbreviations for each CeMV strain or morbillivirus species are as follows: CDV, canine distemper virus; DMV, dolphin morbillivirus; FeMV, feline morbillivirus; GDMV, Guiana dolphin morbillivirus; MeV, measles virus; MBaMV, Myotis bat morbillivirus, PDV, phocine distemper virus; PMV, porpoise morbillivirus; PoMV, porcine morbillivirus; PPRV, peste des petits ruminants virus; PWMV, pilot whale morbillivirus; RPV, rinderpest virus.

The genome of the GDMV_SG_2017 isolate contained six non-overlapping genes in the order N/P-V-C/M/F/H/L, typical of morbillivirus, coding for eight structural and non-structural proteins. The amino acid (aa) length of the six structural proteins encoded by the GDMV_SG_2017 genome was as follows: N, 523 aa; P, 506 aa; M, 335 aa; F, 552 aa; H, 604 aa; L, 2183 aa. Alignment and phylogenetic analysis of complete or near-complete genome sequences available on GenBank demonstrated that the GDMV strain was 74–77.9% (nt) similar to other CeMV strains, with highest nucleotide identity to DMV. The closest sequences (GenBank Acc. nos. MH430932.1 and MH430933.1) are 77.9% nt and 75–95% aa (viral proteins) similar to GDMV_SG_2017 and were isolated from striped dolphins (*Stenella coeruleoalba*) that were stranded in Spain in 1990 during the 1990–1992 Mediterranean Sea morbillivirus epizootics [3,17]. Figure 2 shows the phylogenetic tree obtained using the neighbor-joining method, which includes the available CeMV complete genome sequences and representative sequences of other morbillivirus species. The phylogenetic analysis demonstrated that GDMV_SG_2017 diverges from the other CeMV, forming a separate group closer to a common ancestor.

**Figure 3:**
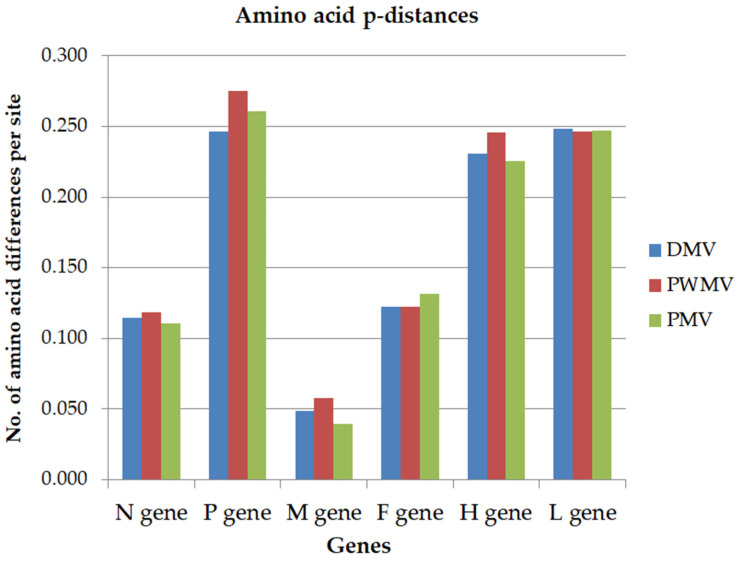
Estimates of evolutionary divergence based on amino acid p-distances per gene between GDMV_SG_2017 and available CeMV sequences representative of DMV (MH430933.1), PWMV (ON492026), and PMV (MH430945.1) strains.

The gene nucleotide identities between GDMV_SG_2017 and other CeMV were 76–77% for the N gene, 79% for the P gene, 79–80% for the M gene, 77–78% for the F gene, 71–72% for the H gene, and 75–76% for the L gene. At the amino acid level, identities between viral proteins of GDMV_SG_2017 and other CeMV were 88–89% aa for the N gene, 72–75% for the P gene, 94–96% aa for the M gene, 87–88% aa for the F gene, 75–77% aa for the H gene, and 75% aa for the L gene. The amino acid diversity (p-distance) across genes of available genomic sequences representative of DMV, PWMV, and PMV strains ranged from 0.039 to 0.275 compared to GDMV_SG_2017 (Figure 3). The representative sequences were selected based on higher homology to GDMV_SG_2017 for the DMV (GenBank Acc. no. MH430933.1) and PMV (GenBank Acc. no. MH430945.1) strains and based on the single sequence available for the PWMV strain (GenBank Acc. no. ON492026.1).

**Figure 4:**
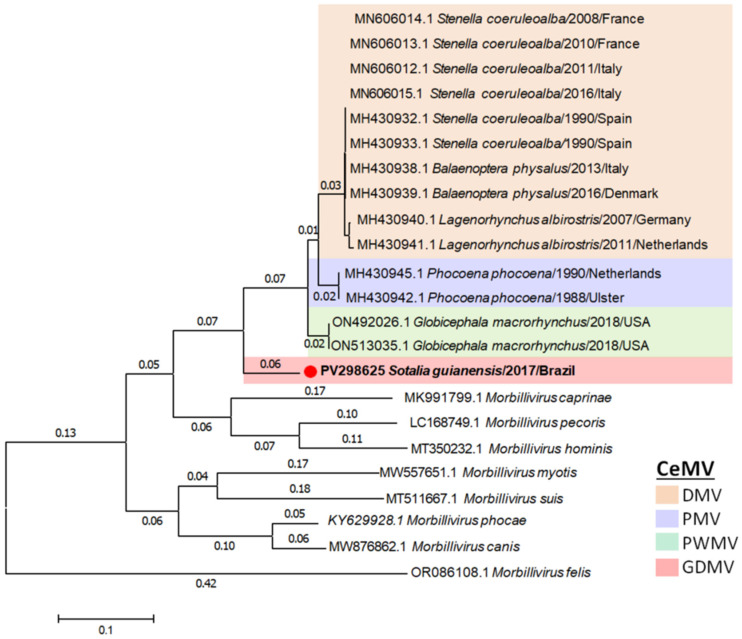
Phylogenetic analysis of complete L protein amino acid sequences of cetacean morbillivirus (CeMV, *Morbillivirus ceti*) isolate found in a stranded Guiana dolphin (*Sotalia guianensis*) from Rio de Janeiro, Brazil, 2017, and those of other previously described morbilliviruses. Complete L protein amino acid sequences were aligned by Clustal W with gap generation penalties of 5 and extension penalties of 1 in both multiple and pairwise alignments. The evolutionary history was inferred by using the Maximum Likelihood method and JTT matrix-based model. The tree is drawn to scale, with branch lengths measured in the number of substitutions per site. This analysis involved 23 amino acid sequences with a total of 2201 positions. The sequence names include GenBank accession number, species of cetacean, year of stranding, and location. The scale bar indicates nucleotide substitutions per site. DMV, dolphin morbillivirus; GDMV, Guiana dolphin morbillivirus; PMV, porpoise morbillivirus; PWMV, pilot whale morbillivirus.

The phylogenetic analysis of the predicted amino acid sequences of N, P, M, F, H, and L proteins displayed similar topologies regarding the position of GDMV_SG_2017 in relation to other CeMV (Supplementary Figure S1). All six trees placed GDMV_SG_2017 as a distinct subgroup with high bootstrap (99–100) supports, and resemble the tree derived from full-length CeMV sequences. A switch on the topology of PMV and PWMV was observed for the M protein. The phylogenetic tree for L protein complete amino acid sequences shows that the genetic distance of GDMV from the node (branch length) was 0.06, which is higher than the distances among any CeMV strain (0.01–0.03) and was comparable to the distances of CDV and PDV (0.06 and 0.05, respectively), which are the closest species within the genus *Morbillivirus* (Figure 4).

## 4. Discussion

In the present work, we successfully isolated a GDMV strain from a Guiana dolphin that died during an unusual mortality event in Brazil during 2017–2018 using Vero.DogSLAMtag cells, and we obtained the nearly complete viral genome by next generation sequencing and gaps closure by Sanger sequencing. These results corroborate the high sensitivity of Vero.DogSLAMtag cell lines for the isolation of CeMV [17,18]. In our study, CPE was observed 24 h after inoculation. Similar results have been reported on the isolation of DMV from archival tissues, with extensive CPE observed after 48 h of incubation [18]. Notably, our previous attempts at GDMV isolation using Vero cells without DogSLAMtag were unsuccessful. Viral isolation was confirmed in the subsequent passages by positive labeling of the cell monolayer with an anti-CDV nucleoprotein monoclonal antibody and detection of GDMV nucleic acids by real time RT-PCR. The reduced number of cell passages (two passages) minimized the potential selection of minor variants or nucleotide changes due to virus adaptation to culture conditions. The viral load obtained on the second cell passage allowed us to perform massive parallel sequencing by NGS directly from the viral RNA without prior PCR amplification, as in previous studies of DMV isolation [18].

The GDMV_SG_2017 genome is the first CeMV-2 genome that has been completely sequenced. The phylogenetic trees of predicted viral protein coding regions placed GDMV_SG_2017 as a separate group of CeMV in all six trees (Figure S1). Although the limited number of available complete genome sequences precludes the analysis of all currently known CeMV strains, partial P gene sequences have successfully been used for global phylogeographic analysis, including all known CeMV strains [17]. As in full genome phylogeny, partial P gene trees cluster GDMV and IPDMV in a separate group diverging from the CeMV-1 strains [25,32].

The nucleotide diversity was determined for each gene by comparing representative sequences of DMV, PWMV, and PWV to GDMV_SG_2017. The P and M genes showed smaller p-distance values and are interpreted as the most conserved genes, while H gene showed larger values that indicate higher nucleotide diversity. The amino acid identities further support the M protein as the most conserved among the GDMV and CeMV-1 strains. The M protein plays a central role in virus assembly, interacting directly with both nucleocapsid and the membrane glycoproteins. Because it is highly conserved across CeMV [33] and other morbilliviruses, the M gene could be a useful target for broad morbillivirus diagnosis. On the other hand, the H gene seems to be among the most variable and might be useful for molecular epidemiologic studies and the characterization of CeMV strains. In the case of measles virus, the H glycoprotein attaches to the receptor on a target cell and most likely stimulates the immune response primarily by triggering the production of neutralizing antibodies [34]. Morbilliviruses use two types of cell surface molecules as receptors: signaling lymphocytic activation molecule (SLAM) and poliovirus receptor-like 4 (PVRL4 or nectin-4), expressed on immune and epithelial cells, respectively [35,36,37]. Because CeMV can infect various cetacean species, including toothed and baleen whales, the CeMV-H protein is postulated to have broader specificity to accommodate more divergent receptor interfaces [38]. Moreover, GDMV may have evolved to infect Southern-hemisphere cetacean species, such as Southern right whales [22] and Guiana dolphins [6,19]. Further characterization of the GDMV H gene could illuminate aspects of tissue tropism, host range, cross-species transmission, and viral mechanisms for immune evasion to overcome host antiviral responses.

According to the International Committee on Taxonomy of Viruses’ (ICTV) report on the family *Paramyxoviridae *(<ictv.global/report/paramxyoviridae>) [39], the current paramyxovirus taxonomic structure and species demarcation criteria are based on a comparison of complete L protein amino acid sequences. In the morbillivirus genus, the L protein is the viral RNA-dependent RNA polymerase responsible for the capping and polyadenylation of viral mRNAs during transcription [40]. The topology and branch lengths >0.03 in a tree with sequences aligned by Clustal W with gap generation penalties of 5 and extension penalties of 1 in both multiple and pairwise alignments, plus an evolutionary history inferred by using the maximum likelihood method and JTT matrix-based model, are used to distinguish a species in the genus *Morbillivirus* [39]. This genetic-based classification indicates that GDMV is a distinct morbillivirus species, given that the branch length between the nearest node and the tip of the branch of GDMV is 0.06. For comparison, the branch lengths of other CeMV strains vary between 0.01 and 0.03, while the branch lengths of CDV and PDV, the closest species within the genus *Morbillivirus*, are 0.06 and 0.05, respectively. Therefore, based on complete L protein amino acid sequences, the branch length of GDMV is comparable to the branch lengths of CDV and PDV. However, the fact that GDMV and CeMV-1 strains affect an apparently similar host range would support the inclusion of GDMV into the CeMV species as a distinct lineage, namely as part of the CeMV-2 lineage. Furthermore, complete L gene sequences from other CeMV strains such as BWMV, IPDMV, and FDMV, which are not currently publicly available, are warranted to provide further insights into the relationship among CeMV strains, as well as their evolutionary histories.

## 5. Conclusions

In conclusion, this work provides the first sequence of the morbillivirus genome in a Guiana dolphin, a virus belonging to the CeMV-2 lineage, which infects cetaceans in the Southern hemisphere. The strain detected shows 77.9% nucleotide homology to DMV, the closest CeMV strain. CeMVs are highly pathogenic, causing acute respiratory disease, immunosuppression, and neurological manifestations. The GDMV strain has been detected in multiple cetacean species along the Brazilian coast and has caused a high mortality outbreak among Guiana dolphins. Complete CeMV sequences derived from both single mortalities and epizootics affecting different cetacean species along the Brazilian coast and other underrepresented geographic regions would be valuable to further assess epidemiologic and evolutionary aspects of cetacean morbilliviruses.

## Figures and Tables

**Table 1:** Position of the gaps in the GDMV genome and list of primers.

Primer No.	nt Gap (nt Position) *	Primer Name	Target Gene	5′ → 3′ Sequence (Sense)	Tm	nt Position *	Template [Reference]
1	985 (1–985)	DMV-1F	3′ leading	ACCARACAAAGYTGGSTARGG (+)	58	1–21	DMV [29]
2		GDMV-1R	N	CCTTGGTTTATTCCCTGGTGT (−)	58	835–855	GDMV [20]
3		DMV-2R	N	AATAGTCATCCGCCTCATCC (−)	57	479–498	DMV [this study]
4		DMV-3F	N	ACCCAGATGTCAGCATCAGA (+)	58	388–407	DMV [this study]
5		CDV-3F	N	ACAGRATTGCYGAGGACYTRT (+)	59	851–871	CDV [30]
6		CDV-4R	N	CARRATAACCATGTAYGGTGC (−)	58	1036–1056	CDV [30]
7	52 (4137–4189)	GDMV-5F	M	CACAAAGGTTCAGGGTGGTC (+)	59	3894–3913	GDMV [this study]
8		GDMV-5R	M	TGAACTCCTGTGGGACTGAA (−)	58	4364–4383	GDMV [this study]
9	150 (5462–5612)	GDMV-7F	F	CAAATCCATTGGGGAAATCT (+)	53	5352–5371	GDMV [this study]
10		GDMV-7R	F	TTGTTAATATCTCCGCCAAGAG (−)	56	5992–6013	GDMV [this study]
11	524 (7387–7911)	GDMV-10F	H	CCAAGACCGAGAAATCATAGAG (+)	56	7136–7157	GDMV [this study]
12		GDMV-10R	H	AACGGTTCATCTTTGTAGCC (−)	56	8015–8034	GDMV [this study]
13	550 (13,375–13,924)	GDMV-13F	L	CCCCTATCATAGAAAAGGATG (+)	43	13,246–13,266	GDMV [this study]
14		GDMV-13R	L	TTTAACTGCTCCTCTCCTGA (−)	45	14,062–14,081	GDMV [this study]

* nt position referenced to DMV (GenBank Accession No. MH430939).

**Table 2:** Combination of primers used in eight RT-PCRs and sequences obtained from the GDMV detected in the present study.

Target Gene	Primer Combinations ^a^	Annealing Temperature	Consensus Obtained (bp)	nt Position in the Reference Genome ^b^
3′ leading and N	1–2 followed by 1–3	55	854	108–787
N	1–2 followed by 4–2	55	388	434–801
N	1–2 followed by 4–6	55	334	555–888
N	5–6	59	122	867–989
M	7–8	55	405	3947–4351
F	9–10	52	512	5408–5919
H	11–12	55	717	7249–7965
L	13–14	52	728	13,301–14,030

^a^ “Primer combinations” refers to the primers described in Table 1. ^b^ nt position referenced to DMV (GenBank Accession No. MH430939).

## Data Availability

All data generated and/or analyzed during the current study are available from the corresponding author on reasonable request.

## Supplementary Materials

The following supporting information can be downloaded at: https://www.mdpi.com/article/10.3390/v17040582/s1. Figure S1. Phylogenetic analysis of complete N, P, M, F, H and L amino acid sequences of GDMV_SG_2017 (GenBank Acc. no. PV298625) and those of other previously described morbilliviruses. The phylogram was generated by the neighbor-joining and p-distance methods with a bootstrap of 1000 replicates and included 21 amino acid sequences. The final datasets contained 516 positions for the N gene, 483 for the P gene, 331 for the M gene, 528 for the F gene, 593 for the H gene, and 2182 for the L gene. The sequence names include GenBank accession number, species of cetacean, year of stranding, and location. The scale bar indicates nucleotide substitutions per site. DMV, dolphin morbillivirus; GDMV, Guiana dolphin morbillivirus; PMV, porpoise morbillivirus; PWMV, pilot whale morbillivirus.
