# Supplementary material for: Complete Genome Sequencing of the Divergent Guiana Dolphin Morbillivirus (GDMV), Brazil
Source: Viruses. 2025 Apr 18;17(4):582. doi: 10.3390/v17040582 (PMC12031208; doi:10.3390/v17040582)
Supplement: Supplementary file 1 [file viruses-17-00582-s001.zip › viruses-3585260-supplementary.pdf]

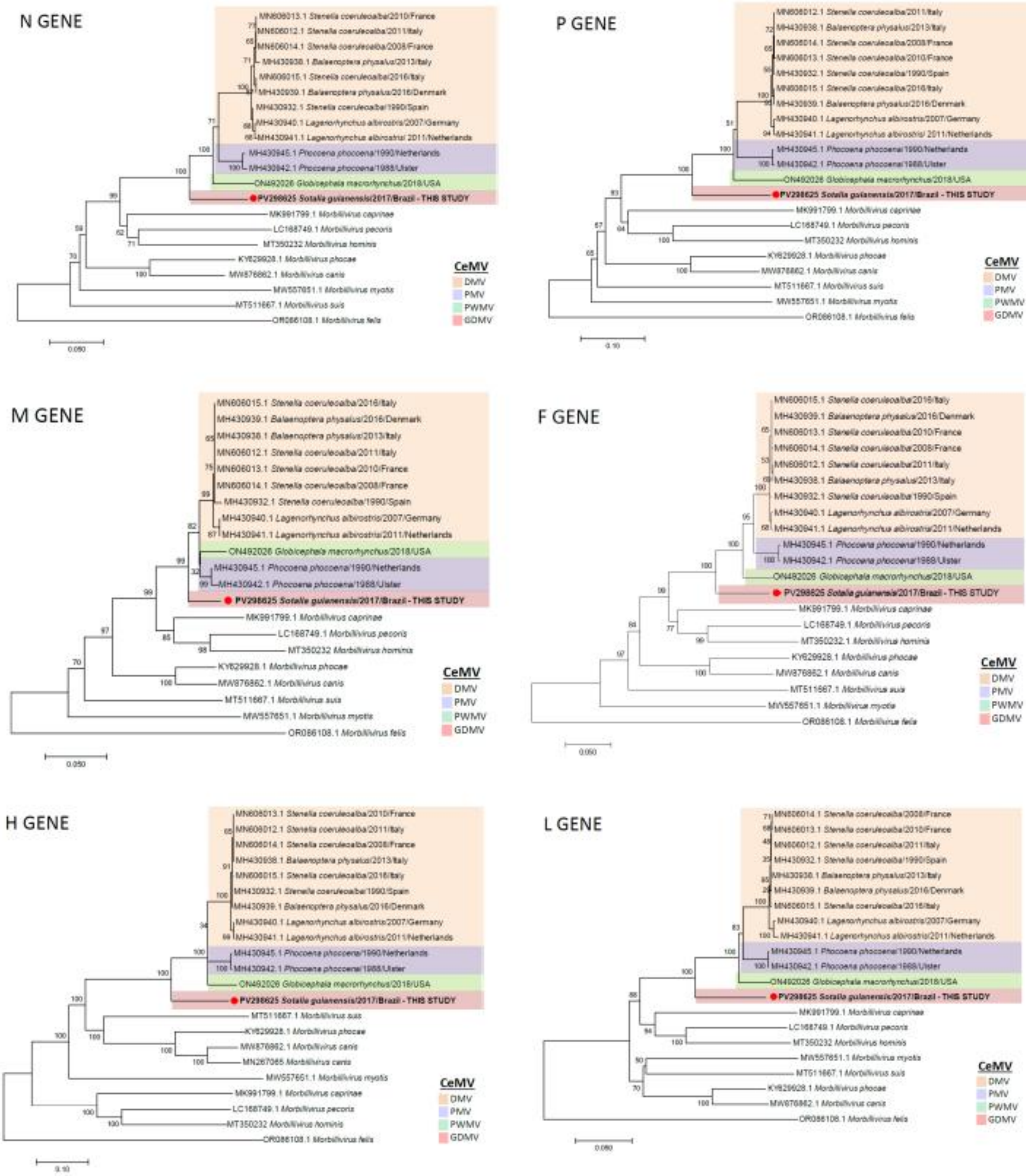

Figure S1. Phylogenetic analysis of complete N, P, M, F, H and L amino acid sequences of GDMV_SG_2017 (GenBank Acc. no. PV298625), and those of other previously described morbilliviruses. The phylogram was generated by the neighbor-joining and p-distance methods, with bootstrap of 1,000 replicates, and included 21 amino acid sequences. The final datasets contained 516 positions for N gene, 483 for P gene, 331 for M gene, 528 for F gene, 593 for H gene, and 2,182 for L gene. The sequence names include GenBank accession number, species of cetacean, year of stranding, and location. The scale bar indicates nucleotide substitutions per site. DMV, dolphin morbillivirus; GDMV, Guiana dolphin morbillivirus; PMV, porpoise morbillivirus; PWMV, pilot whale morbillivirus.
